# Reactive agility training selectively improves choice reaction and footwork-stroke performance in young male table tennis players: a randomized controlled trial

**DOI:** 10.3389/fphys.2026.1903033

**Published:** 2026-09-11

**Authors:** Mingquan Zhang, Wenlong Zhang, Junye Tao, Yana Liu, Rui Jin, Xiao Xu

**Affiliations:** 1 College of Physical Education, Dalian University, Dalian, Liaoning, China; 2 College of Physical Education and Sports, Beijing Normal University, Beijing, China

**Keywords:** choice reaction time, linear mixed-effects model, perception-action coupling, pre-planned agility training, QuickBoard, reactive agility training, table tennis

## Abstract

**Introduction:**

This randomized controlled trial compared the task-specific effects of 6 weeks of QuickBoard-assisted reactive agility training (RAT) and pre-planned agility training (PPAT) in young male table tennis players.

**Methods:**

Forty young male Chinese table tennis players were randomly allocated to RAT (n = 20) or PPAT (n = 20). Both groups trained three times per week for 45 min per session under matched training frequency, planned session duration, and session structure. Pre- and post-intervention assessments included foot speed, choice reaction time, planned change-of-direction performance, table tennis-specific reactive change-of-direction performance, stroke continuity, and an integrated footwork–stroke task requiring accurate ball placement under time pressure. Outcomes were analyzed using linear mixed-effects models including fixed effects for Group, Time, and their interaction.

**Results:**

Significant overall Time effects were observed for all six outcomes (p ≤ .029). Significant Group × Time interactions were identified for choice reaction time (p = .005, d = −0.65) and the Forehand Drive after Backhand Push Test (p = .049, d = 0.55). Bonferroni-adjusted simple-effects analyses showed significant pre-to-post improvements in the RAT group for both outcomes (both adjusted p < .001), whereas the corresponding changes in the PPAT group were not significant. However, adjusted between-group differences at post-test were not statistically significant. No significant Group × Time interactions were observed for foot speed, planned or reactive change-of-direction performance, or the Alternate Counter Test (p ≥ .074).

**Discussion:**

QuickBoard-assisted training was associated with broad improvements over time, whereas the additional benefits of RAT were task-specific. Stimulus-driven and decision-loaded training appeared particularly relevant to choice reaction performance and table tennis tasks requiring the integration of footwork, postural adjustment, and stroke execution under time pressure.

## 1 Introduction

Across competitive sports, successful performance depends not only on physical and technical capacities but also on the rapid integration of perceptual information, cognitive processing, and motor execution (Williams et al., 2011). Research on sport expertise indicates that skilled athletes develop advantages in cue utilization, anticipation, response selection, and domain-specific cognitive processing (Kalén et al., 2021). Meta-analytic evidence further suggests that sport expertise is associated with superior automatic prediction of visual motion and more accurate anticipation of sport-specific actions. These perceptual–cognitive demands are particularly important in fast and open-skill sports, in which environmental information changes continuously and appropriate actions must be selected under severe temporal constraints (Song et al., 2025a; Song et al., 2025b).

Within this broader competitive-sport context, table tennis is a high-intensity, fast-paced, and technique-dominant interceptive open-skill sport in which successful performance depends on the continuous coupling of perception, footwork adjustment, and stroke execution (Faber et al., 2017; Zhang et al., 2023; Chen et al., 2025). In high-level competition, ball speeds are high and rally transitions occur rapidly (Zhang et al., 2018; Zaferanieh et al., 2021); athletes must extract and process information within very short time windows, judge ball direction and placement, and initiate movement rapidly, reposition efficiently, and arrive in a stable posture before ball contact to execute high-quality strokes (Raab et al., 2005; Kondrič et al., 2013; Utama and Widodo, 2021; Tian et al., 2026). Accordingly, performance in table tennis is not determined solely by how fast an athlete can move, but also by how effectively movement is organized to support timely and accurate stroke execution. In this context, reaction ability, short-distance multi-directional footwork, change-of-direction (COD) ability, and footwork–stroke integration are commonly regarded as important contributors to table tennis performance (Castellar et al., 2019; Pradas et al., 2022; Tian et al., 2026).

From a functional perspective, not all agility-related tasks place the same demands on the athlete (Faber et al., 2021; Utama and Widodo, 2021). Pre-planned change-of-direction tasks primarily assess execution-oriented movement under known conditions (McNeil et al., 2021; Young et al., 2021), whereas reactive agility additionally requires cue identification, information processing, and response selection before movement is completed (Young et al., 2015; Čoh et al., 2018; Mackala et al., 2020). This distinction is especially relevant in table tennis, where on-court movement is rarely performed in a fully predictable manner. Players must repeatedly adjust their footwork in response to rapidly changing ball information and use movement not as an end in itself, but as a means of creating a stable and effective hitting position (Castellar et al., 2019; Tian et al., 2026). Therefore, differentiating between pre-planned agility and reactive agility is important when evaluating training strategies intended to transfer to table tennis-specific performance.

Recent table tennis conditioning research has increasingly emphasized that effective agility development should go beyond linear speed or repetitive pre-set footwork. Instead, training should address high-frequency initiation–braking–reacceleration actions, rapid lateral and diagonal repositioning, and the maintenance of stroke preparation quality after movement (Kondrič et al., 2013; Čoh et al., 2018; Zhang et al., 2018; Picabea et al., 2021; Kong and Li, 2023). At the same time, available evidence suggests that improvements in generic movement capacity do not always translate directly into better table tennis performance. In particular, reaction time and displacement measures may relate to performance, but their relationships with stroke outcomes are not consistently stable (Castellar et al., 2019; Chen et al., 2025; Tian et al., 2026). This suggests that training transfer may be expressed more clearly in movement quality, positioning efficiency, and footwork-stroke linkage rather than as an immediate increase in simple count-based indicators (e.g., the number of successful strokes). Within this context, comparing pre-planned agility training (PPAT) with reactive agility training (RAT) has practical and theoretical relevance. Pre-planned agility training (PPAT) can increase training density and execution efficiency through predictable movement patterns, but its informational constraints more closely resemble closed-skill tasks (Šimonek et al., 2016; Sekulic et al., 2017; Mackala et al., 2020). In contrast, reactive agility training (RAT) emphasizes stimulus identification, response selection, and integrated execution under random stimuli, requires the athlete to perceive external cues, select an appropriate response, and execute movement under time pressure, thereby more closely resembling the perception–action demands of competition (Young and Rogers, 2014; Hassan et al., 2022; Steff et al., 2024).

The QuickBoard system is a computerized lower-extremity training and assessment platform rather than an immersive simulation of competitive sport. It combines pressure-sensitive floor pads with a tablet-based interface and can present either predetermined movement sequences or randomized visual cues. The system therefore allows movement direction, cue predictability, and stimulus–response requirements to be manipulated under controlled conditions while recording foot-contact or response-related performance. In the present study, this functionality enabled the same hardware platform to deliver either pre-planned or stimulus-driven footwork exercises (Galpin et al., 2008; Paquette et al., 2017; Engeroff et al., 2018; Zhang et al., 2026). Prior studies suggest that short-term QuickBoard training may improve reaction time and agility-related outcomes, although findings vary across studies and appear to depend on the specificity of the training content and the testing task, consistent with the principle of “specific adaptations to specific stimuli” (Galpin et al., 2008; Niederer et al., 2019; Engeroff et al., 2021; Zhang et al., 2026). However, research on QuickBoard in youth table tennis players remains scarce, and randomized controlled trials directly comparing RAT versus PPAT effects on COD ability, reaction ability, and table tennis-stroke performance are particularly lacking.

Therefore, this study aimed to compare reactive agility training (RAT) and pre-planned agility training (PPAT) delivered through a QuickBoard-assisted program in youth table tennis players. The aims were to: (a) determine whether RAT and PPAT improve COD ability, reaction ability, and table tennis-specific stroke performance; and (b) compare the differential effects of RAT versus PPAT on these performance outcomes. We hypothesized that: (a) both RAT and PPAT would lead to improvements over time across multiple agility-related measures; and (b) RAT and PPAT would produce comparable gains in execution-dominant indicators (e.g., foot speed and selected COD measures), whereas RAT would yield more pronounced benefits on reaction-related tests and sport-specific integrated tasks that impose greater uncertainty and decision-making demands.

## 2 Materials and methods

### 2.1 Participants

An *a priori* sample size calculation was performed using G*Power version 3.1.9.7. Because G*Power does not directly support the planned linear mixed-effects models, the calculation was approximated using a mixed-design ANOVA framework with two groups and two repeated measurements. A small-to-moderate effect size was specified (Cohen’s f = 0.25), with α = 0.05 and power = 0.80 (**Supplementary Figure 1**). The minimum required sample size was 34 participants. To allow for possible attrition, the target sample was increased to 40 participants.

Forty young male Chinese table tennis players aged 15–21 years were recruited from Dalian University Table Tennis Vocational and Technical School for this single-center trial conducted between May and July 2025. Participant characteristics are presented in **Table 1**. Only male athletes were included because the eligible specialized-training cohort available at the school during the recruitment period consisted of male players. The sex composition therefore reflected the accessible training cohort rather than an *a priori* hypothesis that the intervention effects would be specific to male athletes. All participants were in the specialized training stage, a developmental period in which footwork efficiency, repositioning quality, and footwork–stroke coordination remain highly trainable. All athletes had comparable technical levels according to the General Administration of Sport of China (2013). Eligibility criteria were: (a) >=3 years of systematic table tennis training; (b) medically cleared to complete all assessments; (c) right-handed; (d) no neuromuscular disorders or lower-limb injuries in the previous 6 months; and (e) normal or corrected-to-normal vision (excluding color blindness).

**Table 1:** Baseline demographic and training characteristics of participants by group.

Variable	RAT (n=20)	PPAT (n=20)
Age (years)	17.60 ± 1.73	17.65 ± 1.76
Height (cm)	175.52 ± 3.46	172.91 ± 3.53
Weight (kg)	73.73 ± 1.88	73.13 ± 3.95
BMI (kg/m²)	23.95 ± 0.87	24.47 ± 1.41
Training experience (years)	4.81 ± 0.98	4.96 ± 1.25
HRrest (bpm)	54.58 ± 2.26	55.23 ± 1.80
HRmax (bpm)	207.45 ± 1.30	207.59 ± 1.50

Values are presented as mean ± SD. RAT, Reactive Agility Training; PPAT, Pre-Planned Agility Training; BMI, Body Mass Index; HRrest, Resting Heart Rate; HRmax, Maximal Heart Rate.

No significant between-group differences were observed at baseline in body mass, training experience, resting heart rate, or maximal heart rate (**Table 1**). Resting heart rate (HRrest) was measured in the morning after 10 min seated rest. Maximal heart rate (HRmax) was obtained from a maximal incremental test performed during the familiarization week using Yo-Yo IR1, under standardized conditions, and defined as the highest 1-s (or 5-s) heart-rate value recorded during the test. HR-based training zones were expressed as %HRmax.

Before enrollment, all participants were informed of the study procedures, intervention schedule, and testing requirements. Written informed consent was obtained from all participants, and written consent from legal guardians was additionally obtained for minors. The study was conducted in accordance with the Declaration of Helsinki and approved by the institutional ethics committee (Approval No. DLUSPR20250508). All data were anonymized and used exclusively for research purposes.

### 2.2 Trial design

This study was a parallel-group, single-center randomized controlled trial with a 1:1 allocation ratio, employing a Group (RAT vs PPAT) × Time (Pre vs Post) mixed design. The purpose of the design was to compare two QuickBoard-assisted training approaches that differed in cue predictability: one emphasized stimulus-driven reactive footwork (RAT), whereas the other emphasized pre-planned footwork execution (PPAT). Following baseline testing, participants were randomly allocated to reactive agility training (RAT; n = 20) or pre-planned agility training (PPAT; n = 20) using a computer-generated random sequence (SPSS random number generator). The allocation sequence was generated by an investigator who was not involved in recruitment or outcome testing. Group assignments were concealed using sequentially numbered, opaque, sealed envelopes (SNOSE) prepared by an independent staff member and opened only after completion of baseline assessments.

The trial was conducted in the table tennis arena at Dalian University Table Tennis Vocational and Technical School between May and July 2025. Participants completed a 6-week intervention (three sessions per week), with at least 24 h between sessions. Because the two training approaches differed in the predictability of movement cues, blinding of participants and coaches to intervention type was not feasible. To reduce expectancy effects, participants were not informed of the study hypotheses or any presumed superiority of either intervention and were simply told that both programs represented commonly used agility-training approaches. Outcome-data analysts were blinded to group allocation by using coded group labels until completion of the primary analyses. Pre- and post-intervention assessments were scheduled at the same time of day for each participant to reduce potential diurnal variation. No important changes to the protocol or outcomes were made after the trial commenced. This trial was not prospectively registered before participant enrollment. In accordance with the Section Chief Editor’s recommendation, the authors have initiated retrospective registration of the trial in a publicly accessible clinical trial registry. The manuscript will be updated with the registry name, registration number, and date of registration once the retrospective registration process has been completed.

One week before baseline testing, all participants completed a familiarization period to become accustomed to the QuickBoard device, outcome tests, training procedures, and the Firstbeat professional heart-rate monitoring system (Finland; sensor: MOVESENSE; model OP174; product ID: 213830002517). This familiarization period was implemented to reduce learning and novelty-related effects associated with the computerized equipment and testing procedures.

### 2.3 Blinding

Because the two interventions differed in cue predictability and response structure, blinding of participants and coaches to intervention type was not feasible. To reduce expectancy effects, participants were not informed of the study hypotheses or of any presumed superiority of either training approach and were simply told that both programs represented commonly used agility-training methods. Outcome-data analysts were blinded to group allocation using coded group labels until completion of the primary analyses. Pre- and post-intervention assessments were scheduled at the same time of day for each participant to reduce potential diurnal variation.

### 2.4 Trial protocol

**Figure 1:**
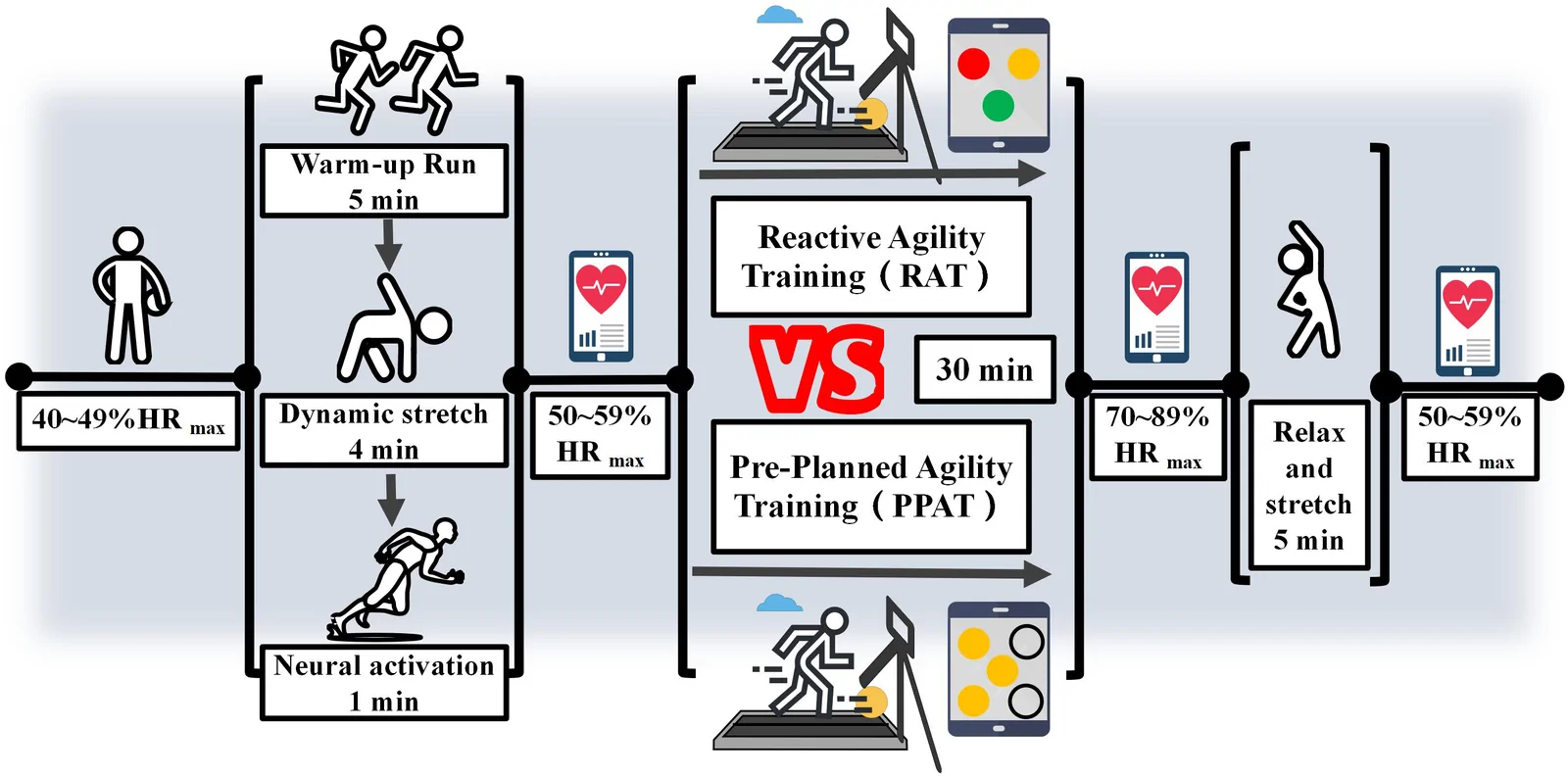
Overview of the experimental protocol.

Both groups completed a 6-week QuickBoard-assisted intervention (three sessions per week; 18 sessions in total; approximately 45 min per session). The two programs were designed to be comparable in planned session duration and general movement-direction exposure, while differing in whether movement responses were stimulus-driven or pre-planned. Each session consisted of a 10-min warm-up, a 30-min main training period, and a 5-min cooldown (**Figure 1**).

During the intervention, participants wore Firstbeat heart-rate monitors to support real-time supervision of exercise intensity during the 30-min main training period. Both groups were prescribed the same target intensity range of 70–89% HRmax, and coaches monitored current heart rate, percentage of HRmax, and the time displayed within the target zone during each session. Work–rest timing was adjusted when necessary to maintain the prescribed intensity while preserving movement quality (Parak and Korhonen, 2013; Palmer et al., 2021; Conte et al., 2025). However, the heart-rate data were used only for real-time session supervision and were not exported in a form that permitted retrospective calculation of mean heart rate or accumulated time in the target zone across the 6-week intervention. Therefore, comparability between the interventions refers to matched training frequency, planned session duration, session structure, target intensity range, and general movement-direction exposure, rather than demonstrated equivalence of physiological internal load.

The training content was developed to reflect table tennis-specific movement demands. In table tennis, successful footwork is not only a matter of moving quickly, but of initiating efficiently, repositioning precisely, and arriving in a stable posture that supports the next stroke. Accordingly, the RAT program was designed to emphasize rapid cue processing, directional judgment, and multidirectional reactive footwork under uncertainty (Young and Farrow, 2013). QuickBoard stimuli were presented in a randomized and unpredictable manner, requiring participants to identify cues quickly and execute the corresponding footwork response. This format was intended to approximate the perceptual–motor demands of responding to changing ball information in table tennis (Raab et al., 2005; Zhang et al., 2023). By contrast, the PPAT program used the same QuickBoard platform but organized movement through fully predictable, pre-planned step sequences. Thus, the emphasis was placed on execution quality, movement rhythm, and footwork organization under known conditions, rather than on stimulus uncertainty or response selection (Young et al., 2015). Participants performed each pattern at maximal controllable speed, with emphasis on step frequency, rhythmic coordination, and table-tennis-relevant footwork execution (Sheppard and Young, 2006).

The QuickBoard system used in this study consisted of five pressure-sensitive floor pads connected wirelessly to a tablet-based interface. Depending on the selected program, the interface displayed either a predetermined sequence of target pads or randomized visual cues corresponding to specific pads. Contact with the designated pad was detected automatically by the system (Galpin et al., 2008; Engeroff et al., 2018, Engeroff et al., 2021; Zhang et al., 2026). In the present trial, QuickBoard served two functions: it was used as the intervention platform for both RAT and PPAT, and it was used as the standardized assessment platform for the Foot Speed Test and Choice Reaction Test. The RAT and PPAT programs used the same physical platform but differed primarily in cue predictability and the requirement for online stimulus identification and response selection.

To preserve a meaningful contrast between the two interventions, PPAT drills were designed to remain broadly comparable to RAT drills in spatial coverage and movement directions while removing stimulus unpredictability. Therefore, the programmed contrast primarily reflected the presence versus absence of stimulus-driven perception–decision demands within table tennis-relevant footwork tasks. Nevertheless, because session-level internal and external training-load data were not available for statistical comparison, residual confounding related to actual training load cannot be fully excluded (Šimonek et al., 2016; Mackala et al., 2020). The detailed training plans for both groups are presented in **Tables 2**, **3**.

**Table 2:** Training content for RAT.

Week	Main training (30 min): QuickBoard drills
Week 1	Double Leg React 10s; React Stability (L/R) 30s; React Stability 0.5 Delay (L/R) 30s; React Stability Iso 35/34 (L/R) 30s; React Stability Iso 25/14 (L/R) 30s; React to Yellow 30s; React To Green 30s; React to Blue/Yellow 30s; React to Green/Red 20s; React Skaters (L/R) 30s; 45 Degree Hip Turns 20s; Hip Turns Jump 20s
Week 2	Double Leg React 30s; React to Green/Yellow/Red 30s; React to Purple/Orange/Green 30s; React to Orange/Yellow 30s; Array: React to Green 20s; Array: React to Red; 30s Array Reaction Foot Fire 20s; React to Green/Red 20s; Array Stability: React to Yellow (L/R) 30s; Array Stability: React to Red (L/R) 30s; 45 Degree Hip Turns 20s; Spider Exercise 30s
Week 3	Double Leg React 30s; 45 Degree Hip Turns 20s; Go/No-Go Congruent 30s; Go/No-Go Incongruent 30s; Stroop Array Congruent 30s; Stroop Array Incongruent 30s; Quick Hop React Iso (L/R) 20s; Quick Hop Go/No-Go: React to Orange (L/R) 20s; Lateral Hop Forward Step (L/R) 20s; Go/No-Go React Stability 15 touches; Hip Turns Jump 20s; Spider Exercise 30s
Week 4	Foot Fire React Front (v1) 20s; Foot Fire React Back (v1) 20s; Foot Fire Go/No-Go Front 20s; Foot Fire Go/No-Go Back 20s; Array Reaction Foot Fire 20s; Backpedal React 20s; 45 Degree Hip Turns 20s; Lateral Hop Forward Step 20s; Single Leg Lateral Cone Hops 20s; Spider Exercise 30s; Hip Turns Jump20s
Week 5	React Skaters (L/R) 30s; Double Leg React 30s; React Skaters Iso (L/R) 30s; 45 Degree Hip Turns 20s; Array React Skater (L/R) 30s; Spider Exercise 30s Array React Skater Iso (L/R) 30s; Crossover Stability React 45 (L/R) 30s; React Stability Iso 35/34 (L/R) 30s; React Stability Iso 25/14 (L/R) 30s; React Stability Iso 35/34 Delay (L/R) 10s; React Stability Iso 25/14 Delay (L/R) 10s;
Week 6	Double Leg React 30s; React to Green/Yellow/Red 30s; Go/No-Go Incongruent 30s; Stroop Array Incongruent 30s; Quick Hop React Iso (L/R) 20s; Foot Fire React Front (v1) (L/R) 20s; Quick Hop Go/No-Go: React to Orange (L/R) 20s; Array Reaction Foot Fire 20s; Backpedal React 20s; 45 Degree Hip Turns 20s; Lateral Hop Forward Step 20s; Spider Exercise 30s

This table summarises the main QuickBoard-based training drills performed during the 30-min RAT training phase in each intervention week. All drills were completed for 3 sets, with 15-s inter-drill rest and a target intensity of 70–89% HRmax. Values expressed in seconds indicate drill duration, whereas “touches” indicates the prescribed number of contacts. L/R, left/right; HRmax, maximal heart rate; Delay, delayed-cue condition.

**Table 3:** Training content for PPAT.

Week	Main training (30 min): QuickBoard drills
Week 1	Ickey Shuffle (Start L/R) 15s; In & Outs (Start L/R) 15s; Out & In 15s; Quicksteps (Forward) 30s; Quicksteps (Backward) 30s; Lateral Hops - Beginner (L/R) 15s; Triangle Hops (L/R) 10s; Figure 8 Hops (L/R) 10s; Slalom Hops 10 s; Tri-Dot Hop (L/R) 10s; Foot Fire 10 s; Hip Pops 15s
Week 2	Lateral Sequence Hops (L/R) 15s; Lateral Hops (L/R) 10s; Z Hops (L/R) 15s; X Hop (L/R) 25 touches; Quick Hops (L/R) 10s; Split Step X Hop 20s; Stagger Step 10s; Gait Speed 15s; Foot Fire Split Step 10s; Foot Fire Staggered 15s; Hip Pops Iso (L/R) 15s; Out & In 15 s
Week 3	Tri-Dot Speed on Board (L/R) 20s; Lateral Tri-Dot Speed (L/R) 20s; Diagonal Quickstep (L/R) 20s; Iso Gait Speed (L/R) 15s; Quicksteps (Forward) 30s; Quicksteps (Backward) 30s; Crossover Intermediate (L/R) 15s; Stagger Step 20s; Slalom Hops 10s; Foot Fire 10s; Foot Fire Split Step 10s; Triangle Hops (L/R) 10s
Week 4	Crossover (Front) (L/R) 15s; Crossover (Back) (L/R) 15s; Crossover Intermediate (L/R) 15s; Crossover Advanced (L/R) 15s; Crossover Stability Seq 45 (L/R) 20s; Lateral Sequence Hops (L/R) 15s; L Drill (L/R) 25 touches; L Drill Inverted (L/R) 25 touches; Z Hops (L/R) 15s; Gait Speed 15s; Foot Fire Staggered 15s; Hip Pops 15s;
Week 5	Tri-Dot Speed on Board (L/R) 20s; Crossover Advanced (L/R) 15s; Diagonal Quickstep (L/R) 20s; Lateral Tri-Dot Speed (L/R) 20s; X Hop (L/R) 25 touches; Lateral Hops (L/R) 10s; Stagger Step 20s; Split Step X Hop 20s; Quick Hops (L/R) 10s; Slalom Hops 10s; Foot Fire Split Step 10s; Hip Pops Iso (L/R) 15s
Week 6	Crossover Stability Seq 45 (L/R) 20s; Crossover Advanced (L/R) 15s; Crossover Intermediate (L/R) 15s; L Drill Inverted (L/R) 25 touches; Lateral Sequence Hops (L/R) 15s; L Drill (L/R) 25 touches; Z Hops (L/R) 15s; Tri-Dot Speed on Board (L/R) 20s; Iso Gait Speed (L/R) 15s; Quicksteps (Backward) 30s; Quicksteps (Forward) 30s; Foot Fire Staggered 15 s

This table summarises the main QuickBoard-based training drills performed during the 30-min PPAT training phase in each intervention week. All drills were completed for 3 sets, with 15-s inter-drill rest and a target intensity of 70–89% HRmax. Values expressed in seconds indicate drill duration, whereas “touches” indicates the prescribed number of contacts. L/R, left/right; HRmax, maximal heart rate.

### 2.5 Outcomes measurement

Assessments were conducted before and after the 6-week intervention at the same time of day for each participant. The testing order was standardized. Participants completed a familiarization session during the week before baseline testing to reduce learning effects associated with the QuickBoard system, testing procedures, and heart-rate monitoring equipment. For each outcome, three trials were performed. The best trial was retained for analysis: fastest time for time-based tests and highest score, count, or point total for performance-based tests. Rest intervals were standardized within each test.

#### 2.5.1 Foot speed test

The Foot Speed (FS) Test was used to assess the capacity for rapid, high-frequency lower-limb adjustments, which form an important physical basis for short-distance footwork regulation in table tennis. The test was performed using the QuickBoard system (test–retest reliability: ICC = 0.89) (Galpin et al., 2008). The device connects to an iPad via Bluetooth and includes five pressure-sensitive pads (upper-right, upper-left, lower-right, lower-left, and center).

Participants stood with the right and left foot positioned over two designated target pads. Following a standardized 5-s countdown, they were instructed to perform alternating rapid foot taps on the two pads for 10 s, while avoiding jumping (**Supplementary Figure 2**). The software automatically recorded the total number of foot contacts. Each participant completed three trials with 60 s of rest between trials, and the highest score was retained for analysis.

#### 2.5.2 Choice reaction test

The choice reaction test was used to assess visual stimulus recognition and rapid stimulus–response mapping, both of which are relevant to the initial response demands of table tennis footwork. The test was conducted on the QuickBoard platform (ICC = 0.89) (Galpin et al., 2008).

Participants stood in a neutral position with both feet centered and ready to respond. After a standardized 5-s countdown, randomized visual stimuli were displayed on the iPad, and participants were required to step on the corresponding pressure pad as quickly as possible before returning to a neutral stance between stimuli (**Supplementary Figure 3**). Trials were performed within a fixed 10-s test window, and the system calculated the mean reaction time (s) across correct responses during that period. Three trials were performed with 60 s of rest, and the best performance (lowest mean reaction time) was retained for analysis.

#### 2.5.3 Modified agility T-test

The Modified Agility T-test was used to assess pre-planned change-of-direction ability. In the context of table tennis, this test reflects execution-oriented directional movement capacity under known movement conditions, rather than stimulus-driven reactive repositioning (Sassi et al., 2009).

The MAT (**Supplementary Figure 4**) followed the standard T-test movement pattern with reduced total distance to better suit the short-distance directional demands relevant to youth racket-sport movement. The total distance covered was 20 m rather than 36.56 m, while the number of directional changes was retained. Participants started at cone A, sprinted to cone B, shuffled to cone C, moved across to cone D, shuffled back to B, and then backpedaled to the starting point at A. Performance time (s) was recorded using Smart Speed timing gates (Fusion Sport, Coopers Plains, Australia). Each participant completed three trials, and the fastest time was retained for analysis.

#### 2.5.4 Reactive agility test for table tennis

The table tennis-specific Reactive Agility Test was used to assess stimulus-driven directional response ability, reflecting the capacity to perceive external cues, make directional decisions, and execute rapid repositioning under uncertain conditions (ICC = 0.955) (Utama and Widodo, 2021). This task was included to better represent the reactive component of on-court movement in table tennis.

**Figure 2:**
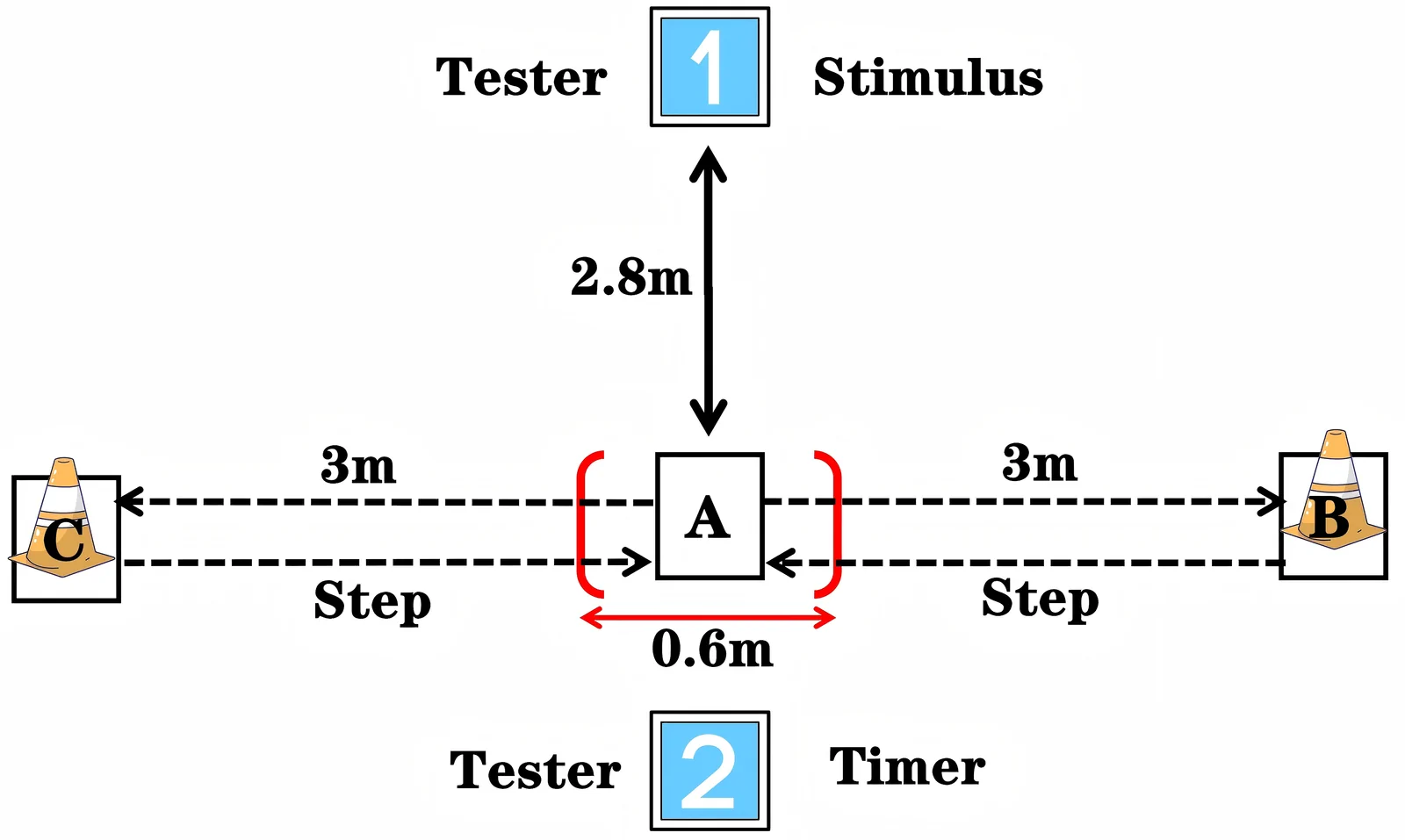
Table tennis reactive agility test.

The participant stood in Box A and focused on the tester’s hands holding the signal flags. When the tester raised the left flag, the participant stepped to the right toward Cone B, touched it, and returned rapidly to Box A. When the right flag was raised, the participant moved to Cone C and returned in the same way (**Figure 2**). If either foot crossed the red line during movement, the tester immediately delivered the next stimulus. A total of six randomized signals were presented (three left and three right). To reduce measurement error, timing began with the first flag stimulus and ended when both feet had fully returned inside Box A after the sixth signal. The same tester administered all trials using a consistent hand position and stimulus distance. Performance time (s) was recorded using Smart Speed timing gates (Fusion Sport, Coopers Plains, Australia). Each participant completed three trials, and the fastest time was retained for analysis.

#### 2.5.5 Alternate counter test

**Figure 3:**
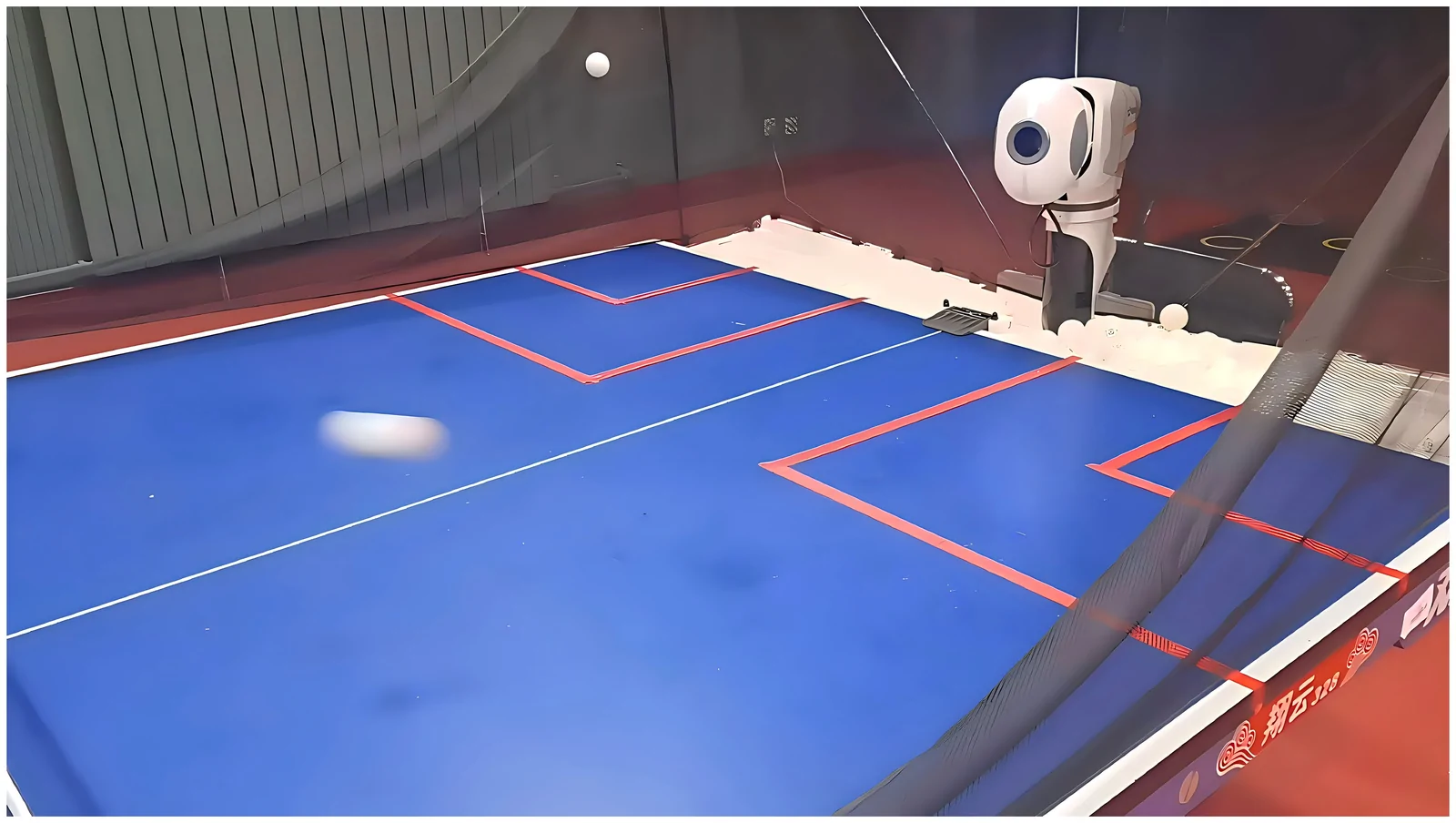
The spatial relationship between the table tennis table and the ball-feeding machine. Note: Table tennis-specific stroke performance setup showing the ball-feeding machine, table, and marked scoring zones used for the stroke performance tests.

The Alternate Counter Test was used to assess table tennis-specific stroke continuity, reflecting the ability to maintain rapid and stable alternating forehand and backhand execution under repetitive rally-like conditions (Purashwani et al., 2010). The DHS D40+ (3-star) table tennis ball was used for testing, and a Youyou X3 PRO ball-feeding machine (Chongqing Yile Youyou Technology Development Co., Ltd., China) was used to deliver the balls (**Figure 3**). Balls were delivered with topspin at a spin rate of 30 revolutions per second, a ball speed of 6 m/s, a launch angle of 10°, and a delivery rate of 70 balls per minute.

Following a standardized warm-up and familiarization, participants completed a 1-min alternating forehand–backhand hitting task on the left half of the table. After the start signal, the ball-feeding machine delivered balls continuously, and the number of successful strokes was recorded. Each participant completed three trials separated by 5 min of rest, and the highest successful-stroke count was retained for analysis.

#### 2.5.6 Forehand drive after backhand push test

The Forehand Drive after Backhand Push Test was used to assess table tennis-specific integrated performance, particularly the ability to reposition efficiently, organize body position after footwork, and execute accurate stroke placement under time pressure (Purashwani et al., 2010).

**Figure 4:**
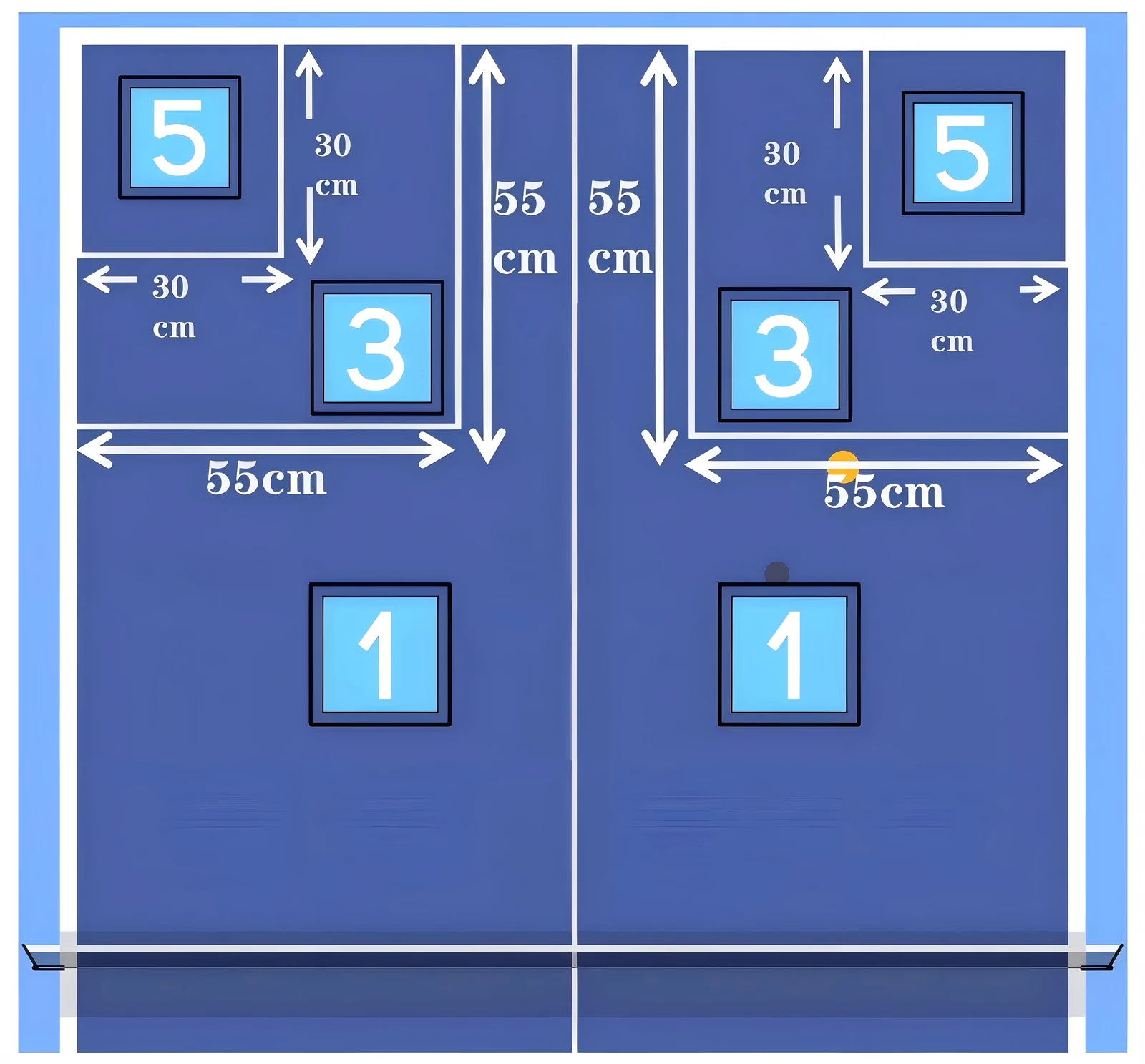
Scoring-zone layout for the Forehand Drive after Backhand Push Test.

The test was conducted on a table tennis table marked with three scoring zones (**Figure 4**). Two target areas measuring 30 × 30 cm were marked at both corners of one half of the table and assigned 5 points. Adjacent to these, two larger 55 × 55 cm zones were marked and assigned 3 points, whereas all remaining areas within the half-table were designated as 1 point. During testing, a camera recorded the entire scoring area to facilitate subsequent score determination. The ball-feeding machine alternately delivered balls to the left and right sides (**Figure 3**), and the participant was required to execute a forehand drive with lateral footwork after first performing a backhand push from the left corner, with each sequence completed within 1 min. The ball-delivery parameters were as follows: topspin, 30 revolutions per second, ball speed of 6 m/s, launch angle of 10°, and delivery rate of 60 balls per minute.

Each participant completed three trials. Trials were recorded using a camera (SONY Alpha 7 III) to capture the landing point of each ball. Videos were then downloaded to a computer and analyzed using Kinovea (www.Kinovea.org), which permits frame-by-frame analysis with an accuracy of 0.02 s. Scoring was based on the landing location of the ball within the marked zones, and the highest score among the three trials was retained as the participant’s final performance score. To determine the reliability of the Kinovea-based video scoring procedure, all recorded trials from the Forehand Drive after Backhand Push Test (i.e., pre- and post-test videos from 40 participants) were independently scored by two assessors using the same predefined scoring criteria. Before formal scoring, both assessors were familiarised with the scoring rules and zone definitions. To reduce potential bias, the two assessors were blinded to each other’s ratings during the reliability assessment. Inter-rater reliability was evaluated using the intraclass correlation coefficient (ICC[2,1]) with 95% confidence intervals.

### 2.6 Statistical analysis

Observed participant characteristics and outcome values are presented descriptively as mean ± standard deviation (M ± SD). All statistical analyses were conducted using R (version 4.4.1; R Core Team, 2024) (Lüdecke et al., 2020). Baseline demographic characteristics and performance outcomes were compared between groups using independent-samples t-tests (**Tables 1**, **4**).

**Table 4:** Descriptive statistics (mean ± SD), baseline between-group differences, and pre-post changes (Δ and Δ%) for all outcomes in the RAT and PPAT groups.

Outcome (unit)	Pre				Post		Δ (Post - Pre)	
	RAT (n=20)	PPAT (n=20)	Baseline difference (RAT-PPAT), 95% CI	p-value baseline	RAT (n=20)	PPAT (n=20)	RAT Δ(Δ%)	PPAT Δ(Δ%)
Foot Speed Test (reps)	109.20 ± 6.44	108.20 ± 9.58	1.00 [−4.23, 6.23]	0.70	114.05 ± 7.04	113.30 ± 6.74	4.85 (+4.44%)	5.10 (+4.71%)
Choice Reaction Test (s)	0.71 ± 0.08	0.71 ± 0.10	0.003 [−0.05, 0.06]	0.92	0.65 ± 0.06	0.70 ± 0.07	−0.06 (−8.45%)	−0.01 (−1.41%)
Modified Agility T-test (s)	6.69 ± 0.20	6.63 ± 0.25	0.06 [−0.08, 0.21]	0.40	6.50 ± 0.21	6.55 ± 0.25	−0.19 (−2.84%)	−0.08 (−1.21%)
Reactive Agility Test (s)	15.52 ± 0.76	15.16 ± 0.71	0.36 [−0.11, 0.83]	0.13	15.14 ± 0.80	15.01 ± 0.61	−0.38 (−2.45%)	−0.15 (−0.99%)
Alternate Counter Test (counts)	54.75 ± 6.63	54.45 ± 5.49	0.30 [−3.60, 4.20]	0.88	58.10 ± 4.82	54.90 ± 3.75	3.35 (+6.12%)	0.45 (+0.83%)
Forehand Drive after Backhand Push Test (points)	121.20 ± 26.05	119.80 ± 19.37	1.40 [−13.30, 16.10]	0.85	138.15 ± 29.20	124.20 ± 20.09	16.95 (+13.98%)	4.40 (+3.67%)

Observed pre- and post-intervention values are presented as mean ± SD. Baseline between-group differences (RAT − PPAT) with 95% confidence intervals (CIs) and corresponding baseline p-values were obtained using two-sided Welch’s independent-samples t-tests. Change scores were calculated as Post − Pre, and percentage change was calculated as (Post − Pre)/Pre × 100%. All values are rounded to two decimals; percentage changes were computed using unrounded means prior to rounding. For time-based outcomes (s), negative change values indicate improvement (faster performance; lower is better), whereas for reps/counts/points outcomes, positive change values indicate improvement (higher is better).

For each outcome, one value per participant at each assessment time point was entered into the analysis. The best performance across the three trials was retained, defined as the fastest time for time-based tests and the highest number of repetitions, successful strokes, or points for performance-based tests. Training effects were examined using linear mixed-effects models (LMMs). Each model included Group (RAT vs PPAT), Time (Pre vs Post), and the Group × Time interaction as categorical fixed effects, together with a participant-specific random intercept to account for the dependence between repeated observations from the same participant. Models were fitted using restricted maximum likelihood estimation with the lme4 package, and denominator degrees of freedom were estimated using Satterthwaite’s approximation implemented in lmerTest (Bates et al., 2015). Model assumptions were evaluated through visual inspection of residual diagnostic plots, including the distribution of residuals and the relationship between fitted values and residuals, to assess normality and homoscedasticity. The primary inferential effect was the Group × Time interaction. Fixed-effect tests are reported using F-statistics, denominator degrees of freedom, and p-values.

Estimated marginal means (EMMs) and model-based contrasts were obtained using the emmeans package. When a significant Group × Time interaction was identified, Bonferroni-adjusted simple-effects analyses were performed to clarify the source of the interaction. Specifically, pre-to-post changes were examined separately within the RAT and PPAT groups, and between-group differences were examined separately at the pre- and post-test assessments. Bonferroni adjustments were applied separately to the two within-group time contrasts and the two between-group contrasts for each outcome. Simple-effects results are reported as estimated mean differences with corresponding standard errors (SEs), Bonferroni-adjusted confidence intervals, t-statistics, and adjusted p-values.

Interaction effect sizes were quantified using Cohen’s d based on the between-group difference in model-estimated pre-to-post change. Cohen’s d was calculated as the model-estimated between-group difference in pre-to-post change divided by the pooled baseline standard deviation. Effect sizes were interpreted as trivial (< 0.20), small (0.20–0.49), moderate (0.50–0.79), or large (≥ 0.80) (Cohen, 1989). Statistical significance was set at p <.05 (Middel and Van Sonderen, 2002).

For the Kinovea-based scoring procedure used in the Forehand Drive after Backhand Push Test, inter-rater reliability was assessed using the intraclass correlation coefficient (ICC[2,1]), based on a two-way random-effects model, absolute agreement, and single measurements. ICC estimates are reported with 95% confidence intervals.

## 3 Results

### 3.1 Participant compliance and baseline equivalence

A total of 40 athletes were assessed for eligibility, and no participants were excluded from the analysis. Forty participants were randomized to the RAT group (n = 20) or the PPAT group (n = 20). All participants completed all 18 prescribed training sessions, corresponding to 100% intervention adherence. No training-related injuries or adverse events were reported during the intervention period. Baseline comparisons showed no statistically significant between-group differences in demographic characteristics or performance outcomes (all p > 0.05; **Tables 1** and **4**). Furthermore, all subsequent models converged satisfactorily without warnings.

### 3.2 Foot speed ability

**Figure 5:**
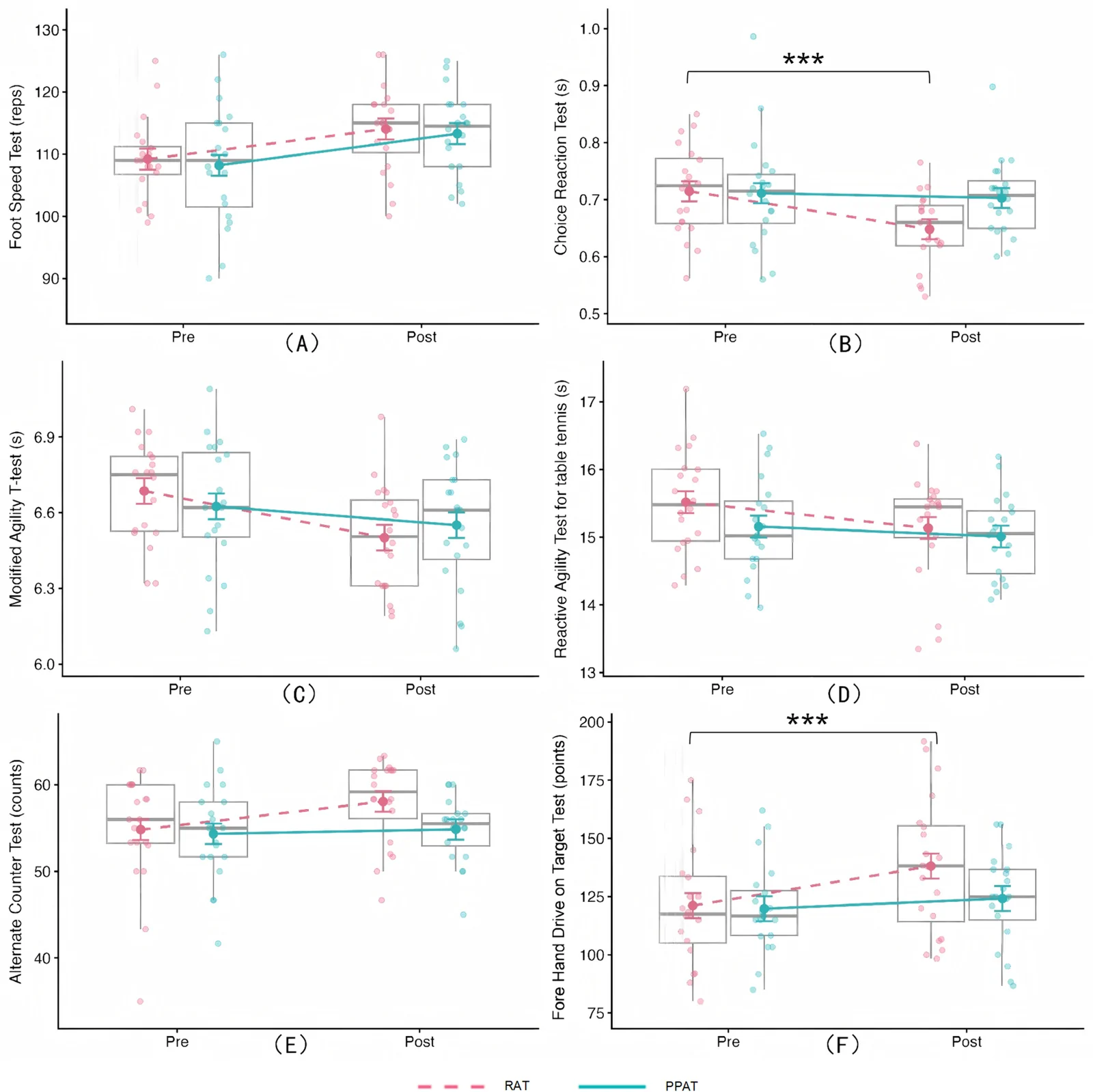
Pre-to-post changes in agility-related outcomes and table tennis-specific stroke performance in the RAT and PPAT groups. **(A)** Foot speed test (repetitions). **(B)** Choice reaction test (s). **(C)** Modified agility T-test (s). **(D)** Table tennis-specific reactive agility test (s). **(E)** Alternate counter test (successful strokes). **(F)** Forehand drive after backhand push test (points). Higher values indicate better performance in **(A, E, F)**, whereas lower values indicate better performance in **(B–D)**. Dots represent individual observations, and boxes show the interquartile range and median. Red dashed and blue solid lines connect the group means for RAT and PPAT, respectively. Asterisks indicate significant Bonferroni-adjusted within-group pre-to-post comparisons: ***p <.001. Only simple-effects comparisons following a significant Group × Time interaction are shown.

Linear mixed-effects modeling showed a significant Time effect (F_(1, 38)_ = 12.10, p = .001, β = −2.49, SE = 0.72, t_(38)_ = −3.48, 95% CI [−3.94, −1.04]), indicating that foot speed values were higher at post-test than at pre-test (**Table 5**; **Figure 5A**). Neither the Group main effect (F_(1, 38)_ = 0.21, p = .650, β = 0.44, SE = 0.96, t_(38)_ = 0.46, 95% CI [−1.50, 2.37]) nor the Group × Time interaction (F_(1, 38)_ = 0.01, p = .931; β = 0.06, SE = 0.72, t_(38)_ = 0.09, 95% CI [−1.39, 1.51], Cohen’s d = −0.03) was significant, indicating comparable pre-post improvements between the RAT and PPAT groups.

**Table 5:** Linear mixed-effects model fixed effects for all outcomes (Group, Time, and Group × Time).

Outcome (unit)	Group		Time		Group × Time		
	F	P	F	P	F	P	Cohen’s d
Foot Speed Test (reps)	0.21	0.650	12.10	0.001	0.01	0.931	−0.03
Choice Reaction Test (s)	1.27	0.267	14.51	<.001	8.70	0.005	−0.65
Modified Agility T-test (s)	0.01	0.930	18.59	<.001	3.37	0.074	−0.49
Reactive Agility Test (s)	1.34	0.254	8.84	0.005	1.75	0.194	−0.32
Alternate Counter Test (counts)	1.63	0.209	5.13	0.029	2.70	0.108	0.48
Forehand Drive after Backhand Push Test (points)	1.22	0.276	11.92	0.001	4.12	0.049	0.55

Linear mixed-effects models were specified as: Outcome ~ Group × Time + (1 | Subject) and fitted using restricted maximum likelihood (REML). Type III tests with Satterthwaite degrees of freedom (df) were used for fixed effects. Cohen’s d represents the standardized between-group difference in pre-post change (ΔΔ) based on model-estimated marginal means. Cohen’s d was calculated as the model-estimated between-group difference in pre-to-post change divided by the pooled baseline standard deviation. For time-based outcomes (s; lower is better), negative d indicates a greater reduction (improvement) in the RAT group relative to the PPAT group; for reps/counts/points outcomes (higher is better), positive d indicates greater improvement in the RAT group. F-statistics and p-values are derived from the fixed-effect tests. Where model coefficients are reported in the text, SE represents the standard error of the corresponding model-estimated coefficient rather than the SD of the observed outcome values. F-statistics and Cohen’s d values are rounded to two decimal places; p-values are rounded to three decimal places.

### 3.3 Reaction ability

A significant Group × Time interaction was observed for the Choice Reaction Test, F_(1, 38)_ = 8.70, p = .005, Cohen’s d = −0.65, indicating that the magnitude of pre-to-post change differed between the RAT and PPAT groups (**Table 5**; **Figure 5B**). Bonferroni-adjusted simple-effects analyses showed that reaction time decreased significantly from pre- to post-test in the RAT group (estimated Post − Pre difference = −0.07 s, SE = 0.01, Bonferroni-adjusted CI [−0.10, −0.03], t_(38)_ = −4.78, adjusted p <.001), whereas the corresponding change in the PPAT group was not significant (estimated Post − Pre difference = −0.01 s, SE = 0.01, Bonferroni-adjusted CI [−0.04, 0.02], t_(38)_ = −0.61, adjusted p >.999).

The groups did not differ significantly at pre-test (estimated RAT − PPAT difference = 0.003 s, SE = 0.03, Bonferroni-adjusted CI [−0.06, 0.06], t_(38)_ = 0.12, adjusted p >.999). Although the RAT group demonstrated a lower reaction time than the PPAT group at post-test, the between-group difference did not remain statistically significant after Bonferroni adjustment (estimated RAT − PPAT difference = −0.06 s, SE = 0.03, Bonferroni-adjusted CI [−0.113, 0.004], t_(38)_ = −2.19, adjusted p = .069). The overall Time main effect was significant, F_(1, 38)_ = 14.51, p <.001, whereas the Group main effect was not significant, F_(1, 38)_ = 1.27, p = .267. Collectively, these findings indicate that the significant interaction was primarily attributable to the marked pre-to-post improvement observed in the RAT group.

### 3.4 COD ability

COD ability was assessed using modified agility t-test (planned COD) and the table tennis-specific reactive agility test (reactive COD). A significant Time effect was observed for the modified agility t-test (F_(1, 38)_ = 18.59, p <.001; β = 0.07, SE = 0.02, t_(38)_ = 4.31, 95% CI [0.03, 0.10]) and the reactive agility test (F_(1, 38)_ = 8.84, p = .005, β = 0.13, SE = 0.05, t_(38)_ = 2.97, 95% CI [0.04, 0.22]), indicating that completion times were lower at post-test than at pre-test for both COD measures (**Tables 4**, **5**; **Figures 5C, D**). The Group main effect was not significant for either outcome (Modified Agility T-test: F_(1, 38)_ = 0.01, p = .930, β = 0.01, SE = 0.03, t_(38)_ = 0.09, 95% CI [−0.06, 0.07]; Reactive Agility Test: F_(1, 38)_ = 1.34, p = .254; β = 0.12, SE = 0.11, t_(38)_ = 1.16, 95% CI [−0.09, 0.33]). The Group × Time interaction was not significant for either test (Modified Agility T-test: F_(1, 38)_ = 3.37, p = .074, β = 0.03, SE = 0.02, t_(38)_ = 1.84, 95% CI [−0.01, 0.06], Cohen’s d = −0.49; Reactive Agility Test: F_(1, 38)_ = 1.75, p = .194, β = 0.06, SE = 0.05, t_(38)_ = 1.32, 95% CI [−0.03, 0.15], Cohen’s d = −0.32), indicating no statistically reliable between-group difference in the magnitude of pre-post change. Visual inspection of **Figures 5C, D** suggests a larger pre-post reduction in the RAT group than in the PPAT group for both tests, although these between-group differences were not supported statistically.

### 3.5 Table tennis-specific stroke performance

The Kinovea-based scoring procedure for the Forehand Drive after Backhand Push Test demonstrated excellent inter-rater reliability (ICC[2,1] = 0.98, 95% CI [0.97, 0.99]). For the Alternate Counter Test, a significant Time main effect was observed, F_(1, 38)_ = 5.13, p = .029, indicating that performance scores were higher at post-test than at pre-test (**Tables 4**, **5**; **Figure 5E**). Neither the Group main effect, F_(1, 38)_ = 1.63, p = .209, nor the Group × Time interaction, F_(1, 38)_ = 2.70, p = .108, Cohen’s d = 0.48, was statistically significant, indicating comparable pre-to-post changes in the RAT and PPAT groups. For the Forehand Drive after Backhand Push Test, a significant Group × Time interaction was observed, F_(1, 38)_ = 4.12, p = .049, Cohen’s d = 0.55, indicating that the magnitude of pre-to-post improvement differed between the RAT and PPAT groups (**Tables 4**, **5**; **Figure 5F**). Bonferroni-adjusted simple-effects analyses showed that performance scores increased significantly from pre- to post-test in the RAT group (estimated Post − Pre difference = 16.95 points, SE = 4.37, Bonferroni-adjusted CI [6.75, 27.15], t_(38)_ = 3.88, adjusted p <.001), whereas the corresponding increase in the PPAT group was not significant (estimated Post − Pre difference = 4.40 points, SE = 4.37, Bonferroni-adjusted CI [−5.80, 14.60], t_(38)_ = 1.01, adjusted p = .641).

The groups did not differ significantly at pre-test (estimated RAT − PPAT difference = 1.40 points, SE = 7.60, Bonferroni-adjusted CI [−16.34, 19.14], t_(38)_ = 0.18, adjusted p >.999). At post-test, the RAT group had a higher estimated score than the PPAT group, but the between-group difference was not statistically significant (estimated RAT − PPAT difference = 13.95 points, SE = 7.60, Bonferroni-adjusted CI [−3.79, 31.69], t_(38)_ = 1.84, adjusted p = .148). The overall Time main effect was significant, F_(1, 38)_ = 11.92, p = .001, whereas the Group main effect was not significant, F_(1, 38)_ = 1.22, p = .276. Collectively, these findings indicate that the significant interaction was primarily attributable to the marked pre-to-post improvement observed in the RAT group rather than to a statistically significant between-group difference at either assessment.

## 4 Discussion

This study compared the effects of a 6-week QuickBoard-assisted reactive agility training program and a pre-planned agility training program on agility-related and table tennis-specific performance in young trained male table tennis players. The main finding was that both training programs were associated with significant improvements in foot speed, planned change-of-direction performance, table tennis-specific reactive change-of-direction performance, and table tennis-specific stroke outcomes. However, the additional benefit of reactive agility training was task-specific rather than global, with greater improvements observed only in choice reaction time and the Forehand Drive after Backhand Push Test. Between-group differences were not statistically confirmed for foot speed, the modified agility T-test, the table tennis-specific reactive agility test, or the Alternate Counter Test, indicating that execution-dominant outcomes may respond similarly to both reactive and pre-planned QuickBoard-based training. These findings suggest that QuickBoard-assisted training can improve foundational footwork and directional movement capacities, whereas reactive constraints appear to provide additional benefits when the task requires stimulus identification, response selection, and footwork–stroke integration under uncertainty. This interpretation is particularly relevant for table tennis because competitive performance depends not only on moving quickly, but also on perceiving ball information early, selecting an appropriate response, and arriving in a stable position for stroke execution (Raab et al., 2005; Kondrič et al., 2013; Castellar et al., 2019; Tian et al., 2026).

### 4.1 Effects on foot speed and foundational movement capacity

Both groups improved in the QuickBoard foot speed test, and the absence of a significant Group × Time interaction indicates that reactive and pre-planned formats produced comparable gains in this execution-dominant outcome. This result is likely explained by the shared movement structure of the two interventions, because both required high-frequency stepping, rapid foot contacts, short-distance repositioning, and repeated lower-limb coordination under time pressure (Galpin et al., 2008; Donahue et al., 2016). The foot speed test primarily reflects rapid repetitive stepping capacity and motor sequence efficiency rather than perception-based agility or sport-specific decision-making (Sheppard and Young, 2006; Galpin et al., 2008). Therefore, improvements in foot speed should be interpreted as gains in foundational movement capacity rather than as direct evidence of improved competitive agility (Galpin et al., 2008; Young et al., 2015).

In table tennis, rapid foot contacts and short adjustment steps may help athletes reposition within the small playing area and prepare for subsequent strokes, but these movements become performance-relevant only when they are coupled with ball perception and stroke preparation (Kondrič et al., 2013; Castellar et al., 2019; Picabea et al., 2021). The similar improvement in both groups suggests that predictable and unpredictable QuickBoard drills may provide sufficient lower-limb movement stimulus to enhance basic stepping speed in young table tennis players (Galpin et al., 2008; Paquette et al., 2017). However, because the foot speed test uses a constrained and predictable response structure, it cannot capture the perceptual, tactical, and opponent-driven demands that characterize real table tennis movement (Young and Farrow, 2013; Zhang et al., 2018; Tian et al., 2026). These findings reveal that both the RAT and PPAT, when conducted using the QuickBoard device, effectively develop fundamental speed abilities in adolescent table tennis athletes. Although the FS test cannot simulate the perceptual and tactical demands of competition, the enhanced capacity for rapid repetitive footwork remains a crucial physical foundation.

### 4.2 Effects on change-of-direction performance

Both the modified agility T-test and the table tennis-specific reactive agility test improved after training, indicating that QuickBoard-assisted training may enhance short-distance directional movement capacity in young table tennis players (Sassi et al., 2009; Chen et al., 2025). The lack of statistically confirmed between-group differences suggests that the additional decision-making demands in RAT did not produce clearly superior COD adaptations under the present testing conditions (Čoh et al., 2018; Mackala et al., 2020; Morral-Yepes et al., 2023). This finding is consistent with the conceptual distinction between change-of-direction speed and reactive agility, because planned COD tasks primarily assess braking, reacceleration, directional control, and movement technique, whereas reactive agility additionally requires perception and response selection (Sheppard and Young, 2006; Young et al., 2015, Young et al., 2021). Even when movement patterns are similar, pre-planned COD and reactive agility can represent partly independent abilities because the information-processing demands differ across tasks (Čoh et al., 2018; Morral-Yepes et al., 2023).

The modified agility T-test used in this study is a reliable field measure of short-distance directional movement, but it remains a closed-skill test because the direction and sequence of movement are known in advance (Sassi et al., 2009; Young and Farrow, 2013). The table tennis-specific reactive agility test introduced a stimulus-response component, but its outcome was still based on total completion time, which combines perception, decision-making, movement initiation, braking, and reacceleration into a single composite measure (Utama and Widodo, 2021; Young et al., 2021). Because both RAT and PPAT involved repeated multidirectional stepping, braking, and reacceleration, both interventions may have improved the movement-execution components that strongly contribute to COD performance (Brughelli et al., 2008; Chaabene et al., 2018; Dos’ Santos et al., 2018).

The numerical tendency favoring RAT in COD outcomes may indicate a possible benefit of reactive constraints, but the absence of statistical confirmation requires a conservative interpretation (Batterham and Hopkins, 2006; Lakens, 2013). Future studies should separate decision time and movement time during reactive COD tasks to determine whether RAT improves the perceptual-cognitive component, the movement-execution component, or both (McNeil et al., 2021; Young et al., 2021; Morral-Yepes et al., 2023).

### 4.3 Effects on choice reaction ability

The clearest between-group difference was observed for choice reaction time, with RAT producing a larger improvement than PPAT. This result supports the principle of task specificity because RAT repeatedly required athletes to detect random visual cues, select the appropriate response, and execute the corresponding footwork action under time pressure (Young and Farrow, 2013; Freyler et al., 2016). In contrast, PPAT emphasized execution of known movement sequences, which likely improved movement rhythm and stepping efficiency but imposed less demand on stimulus discrimination and response selection (Young et al., 2015; Engeroff et al., 2018). The larger improvement in the RAT group is therefore best interpreted as a task-specific enhancement of visual stimulus–response coupling rather than as a general improvement in all agility-related abilities. This interpretation is supported by open-skill and computerized agility training studies showing that training effects are strongest when the practiced task and the test share similar stimulus type, response structure, and movement requirements (Galpin et al., 2008; Engeroff et al., 2018, Engeroff et al., 2021; Faber et al., 2021; Zhang et al., 2026).

The improvement in choice reaction time may reflect more efficient cue detection, faster stimulus-response mapping, shorter response selection time, or improved initiation of the correct foot response (McNeil et al., 2021; Jothi et al., 2025). Nevertheless, the current test did not decompose stimulus detection time, decision time, and movement initiation time, so the specific component responsible for the improvement cannot be determined. In table tennis, faster choice reaction ability may help athletes initiate repositioning earlier after perceiving incoming ball information, which may increase the likelihood of arriving in balance before stroke execution (Castellar et al., 2019; Pradas et al., 2022; Tian et al., 2026). However, reaction ability alone should not be equated with match performance, because table tennis outcomes also depend on technical skill, tactical decision-making, ball control, spin interpretation, and psychological regulation (Raab et al., 2005; Zhang et al., 2018; Tian et al., 2026).

### 4.4 Table tennis-specific stroke performance

Both table tennis-specific stroke outcomes improved over time, consistent with previous evidence that structured footwork, multiball, and perceptual–motor practice can support stroke execution and skill development in table tennis players (Paul et al., 2011; Cao et al., 2020; Oagaz et al., 2021). However, only the Forehand Drive after Backhand Push Test showed a significant Group × Time interaction, with the RAT group exhibiting a greater pre-to-post improvement than the PPAT group, indicating that transfer from agility training may depend on the informational and motor requirements of the outcome task. The simple-effects results further showed that this interaction was primarily attributable to a significant improvement within the RAT group rather than to a statistically significant between-group difference at post-test, supporting a cautious interpretation of the task-specific training effect. This divergent pattern may be explained by differences in the relative perceptual-decision and action-organization demands of the two stroke tests.

The Alternate Counter Test required participants to execute a stable and predictable sequence of alternating forehand and backhand strokes under standardized ball-feeding conditions. Once the movement rhythm had been established, performance depended predominantly on repeated stroke execution, temporal consistency, racket control, and the maintenance of a continuous hitting pattern (Purashwani et al., 2010; Paul et al., 2011; Cao et al., 2020). Because both the ball-feeding sequence and the required stroke responses were predetermined, the task imposed relatively limited demands on online stimulus discrimination, response selection, and reorganization of the movement–stroke sequence. The shared high-frequency stepping, movement timing, and execution practice incorporated in both RAT and PPAT may therefore have been sufficient to improve performance in this relatively execution-dominant task. Consequently, the limited perceptual-decision load of the Alternate Counter Test may have reduced its sensitivity to the additional stimulus-driven demands included in RAT (Freyler et al., 2016; Engeroff et al., 2021; Faber et al., 2021). In contrast, the Forehand Drive after Backhand Push Test required participants to transition from a backhand push to lateral repositioning and a subsequent forehand drive while regulating body position and directing the ball toward predefined scoring zones. Although the ball-feeding pattern remained standardized and did not reproduce the full uncertainty of an opponent-driven rally, the task imposed greater relative demands on action switching, footwork timing, postural reorganization, and spatial control than the Alternate Counter Test (Raab et al., 2005; Castellar et al., 2019; Tian et al., 2026). Successful performance therefore required participants not only to execute the strokes correctly but also to coordinate the transition between strokes, reposition the lower limbs efficiently, stabilize the body before contact, and adjust the racket trajectory to achieve accurate ball placement (Basiri et al., 2020; Chen et al., 2025; Tian et al., 2026). Compared with the repetitive stroke rhythm required in the Alternate Counter Test, the Forehand Drive after Backhand Push Test placed greater demands on the integration of footwork, postural control, stroke preparation, and placement accuracy under time pressure.

The larger improvement observed in the RAT group may therefore reflect a closer correspondence between the stimulus-driven demands of the intervention and the integrated movement requirements of the Forehand Drive after Backhand Push Test. During RAT, athletes repeatedly identified visual cues, selected an appropriate directional response, initiated movement under temporal pressure, and reorganized foot placement before completing the required action. Such practice may have strengthened the coordination between cue processing, early movement initiation, lateral repositioning, postural stabilization, and subsequent stroke preparation (Raab et al., 2005; Castellar et al., 2019; Tian et al., 2026). These adaptations may have transferred more readily to a task in which movement transition and placement accuracy had to be coordinated within the same action sequence (Oagaz et al., 2021; Tian et al., 2026). By comparison, the lower decision and action-switching demands of the Alternate Counter Test may have made it more responsive to the common execution-related training stimulus received by both groups (Galpin et al., 2008; Paquette et al., 2017; Engeroff et al., 2018; Zhang et al., 2026).

From a table tennis perspective, stroke quality depends partly on whether an athlete reaches an appropriate hitting position early enough to stabilize the body and regulate the racket trajectory before ball contact (Raab et al., 2005; Chen et al., 2025; Tian et al., 2026). Reactive footwork training may therefore enhance the preconditions for effective stroke execution by promoting earlier movement organization, more efficient repositioning, and a more stable posture at contact (Castellar et al., 2019; Pradas et al., 2022; Tian et al., 2026). Improvements in foot placement and control of braking–propulsion transitions may also facilitate the timely preparation of the subsequent stroke after rapid lateral movement (Brughelli et al., 2008; Chaabene et al., 2018; Dos’ Santos et al., 2018; Young et al., 2021). These adaptations may be particularly relevant for placement-based outcomes because improvements in footwork timing and postural control may first be expressed through more consistent ball placement and stroke quality before becoming evident in simpler count-based measures. Nevertheless, this interpretation should remain cautious because both stroke tests were conducted under semi-controlled ball-feeding conditions and did not incorporate opponent deception, unpredictable placement, variable spin, tactical pressure, or continuous interactive rallies (Raab et al., 2005; Young et al., 2015; Zhang et al., 2018; Tian et al., 2026). Standardized testing improves experimental control and repeatability, but it may reduce the representativeness of the perceptual information and response uncertainty encountered during competitive play (Young and Farrow, 2013; Faber et al., 2021; Young et al., 2021). Consequently, the greater improvement observed in the Forehand Drive after Backhand Push Test should not be interpreted as direct evidence of improved match performance. Future studies should therefore incorporate randomized multiball protocols, opponent-referenced response tasks, deceptive or variable placement cues, and match-derived performance indicators to determine whether the task-specific benefits of RAT extend to more representative competitive environments.

### 4.5 Mechanistic interpretation

The overall pattern of results can be explained through the interaction between task specificity, perception–action coupling, and execution-level movement adaptation (Sheppard and Young, 2006; Young and Farrow, 2013; Freyler et al., 2016). Both RAT and PPAT provided repeated exposure to short-distance stepping, braking, reacceleration, and directional repositioning, which may explain the shared improvements in foot speed and COD-related outcomes (Brughelli et al., 2008; Galpin et al., 2008; Dos’ Santos et al., 2018). These adaptations may involve improved intermuscular coordination, more efficient foot placement, better control of braking–propulsion transitions, and enhanced movement economy during repeated directional tasks (Jones et al., 2009; Marshall et al., 2014; Chaabene et al., 2018).

RAT added a perceptual-decision constraint by requiring athletes to respond to unpredictable visual stimuli, which may explain the greater improvement in the choice reaction test. The additional transfer to the Forehand Drive after Backhand Push Test suggests that reactive constraints may also improve the coordination between early movement initiation and subsequent technical execution when the test requires both repositioning and accurate stroke placement. This task-specific pattern is consistent with broader evidence that sport expertise and training adaptations are most evident when perceptual information, response selection, and motor demands resemble those of the target performance context (Young and Farrow, 2013; Freyler et al., 2016; Young et al., 2021).

However, the present outcomes were composite performance measures, and the study therefore cannot determine whether RAT primarily improved perceptual–cognitive processing, movement execution, or the coordination between these components. Future studies should decompose reactive performance into decision time and movement time to identify the specific source of training-related improvements. Decision time could be defined as the interval from stimulus onset to the initiation of the first detectable movement, whereas movement time could be defined as the interval from movement initiation to target contact or task completion (McNeil et al., 2021; Young et al., 2021; Morral-Yepes et al., 2023). A selective reduction in decision time would provide stronger evidence that RAT improves cue identification, stimulus discrimination, and response selection. In contrast, a selective reduction in movement time would suggest that the primary adaptation occurred in movement initiation, stepping efficiency, braking, or reacceleration (Čoh et al., 2018; McNeil et al., 2021; Young et al., 2021; Morral-Yepes et al., 2023). Improvements in both components would support a combined perception–action adaptation, consistent with the view that reactive agility depends on the effective coupling of perceptual information, response selection, and movement execution (Young and Farrow, 2013; Freyler et al., 2016; Young et al., 2021). Integrating synchronized stimulus presentation with force platforms, motion capture, inertial sensors, or high-speed video would permit these components to be distinguished and provide a more precise mechanistic explanation of how reactive agility training influences table tennis-specific performance.

### 4.6 Practical implications

The practical implication of this study is that PPAT and RAT should be used for different training purposes rather than treated as interchangeable agility methods. PPAT may be appropriate when the training goal is to develop movement rhythm, repeated stepping density, basic directional control, and execution consistency under predictable conditions (Galpin et al., 2008; Paquette et al., 2017). RAT may be preferable when the training goal is to improve response speed, visual cue processing, choice reaction ability, and footwork–stroke linkage under uncertainty (Young and Farrow, 2013; Engeroff et al., 2021; Tian et al., 2026; Zhang et al., 2026). For youth table tennis players, QuickBoard-based RAT may function as a bridge between general agility training and representative on-table practice because it combines high-density lower-limb movement with stimulus-response demands.

A practical progression may begin with PPAT to establish movement rhythm and footwork control, progress to RAT to introduce stimulus uncertainty, and then transfer the gains into multiball drills, variable placement tasks, sparring, and constrained match-play. Coaches should emphasize not only moving faster but also perceiving earlier, initiating the first step efficiently, recovering to a neutral position, arriving in balance, and matching footwork rhythm to stroke timing. QuickBoard training should therefore be considered a supplementary conditioning and perceptual–motor tool rather than a substitute for table-based technical-tactical training.

## 5 Limitations

There are some limitations in this study. First, the participants were young male trained table tennis players recruited from a single vocational training setting. The inclusion of only male athletes reflected the composition of the eligible training cohort available during recruitment, but it limits the generalizability of the findings to female athletes, other developmental stages, and elite or professional players. Future studies should recruit both male and female players and examine whether sex moderates the magnitude or pattern of adaptation to reactive and pre-planned agility training. Replication is also required across different developmental stages and in elite or professional populations (Pradas et al., 2022).

Second, related QuickBoard work has highlighted that small samples and constrained designs may yield low statistical power and modest effect sizes (Galpin et al., 2008), underscoring the need for larger trials to detect between-group differences in sport-specific outcomes.

Third, although the two table tennis-specific stroke-performance tests enhanced the sport specificity of the assessment battery, they remain semi-closed assessments with standardized scoring and cannot fully reproduce opponent-driven variability and perception-action coupling in competition. Accordingly, future research should incorporate opponent-referenced or more representative assessments. For example, reactive multi-ball patterns with deceptive cues, sparring-based diagnostics, or match-derived indicators, should not be limited to laboratory testing.

Fourth, the mechanistic interpretation of the findings is limited because reactive agility involves the integrated contributions of perceptual processing, response selection, and movement execution (Young et al., 2021). In the present study, reaction and reactive-agility performance was assessed using composite time- or score-based outcomes that did not distinguish the interval from stimulus onset to movement initiation from the interval between movement initiation and task completion. Consequently, it remains unclear whether the RAT-related improvements were primarily attributable to faster perceptual–decision processes, more efficient movement execution, or adaptations in both components. Future studies should therefore synchronize stimulus presentation with movement-onset detection to separate decision time from movement time and combine these measures with eye-tracking, lower-limb kinematics, force-platform assessment, and ball-tracking data to provide a more precise mechanistic explanation of training-related adaptations.

Fifth, although both interventions were matched for training frequency, planned session duration, session structure, target heart-rate range, and general movement-direction exposure, session-level heart-rate data were not exported for retrospective analysis. Consequently, mean heart rate and accumulated time within the prescribed intensity zone could not be summarized or statistically compared between groups. External-load indicators, such as foot contacts, accelerations, decelerations, and change-of-direction volume, and session-RPE were also not collected. Therefore, the study cannot confirm that the physiological and mechanical training loads were equivalent between groups, and residual load-related confounding cannot be excluded when interpreting the between-group effects.

Finally, the relatively short duration of the study (6 weeks) limits conclusions regarding the long-term retention or sustainability of the observed training effects. Addressing these limitations in future research would further strengthen the interpretation and practical applicability of the findings.

## 6 Conclusion

This exploratory randomized trial showed that 6 weeks of QuickBoard-assisted RAT and PPAT were associated with improvements in footwork speed, change-of-direction performance, and table tennis-specific stroke outcomes in young trained male table tennis players. The additional effect of RAT appeared to be task-specific, with greater gains in choice reaction time and the Forehand Drive after Backhand Push Test. These results suggest that stimulus-driven training may be most useful when performance depends on rapid cue recognition, response selection, and the integration of footwork with stroke execution under time pressure. In practice, PPAT may be used to reinforce movement rhythm, stepping density, and basic directional control, whereas RAT may be more appropriate when the training goal is to improve reactive ability and perception–action coupling. For table tennis players, effective footwork training should therefore emphasize not only movement speed, but also earlier information pickup, efficient response selection, and balanced preparation for the next stroke.

## Data Availability

The raw data supporting the conclusions of this article will be made available by the authors, without undue reservation.

## Supplementary material

Supplementary Figure 1 G*Power calculation.

Supplementary Figure 2 QuickBoard Foot Speed Test setup and tablet interface. **(A)** An adult participant performing the 10-s alternating foot-tapping task on two designated pressure-sensitive pads while facing the tablet display. **(B)** The QuickBoard tablet interface used to present the Foot Fire 10 Seconds protocol and record left- and right-foot contacts. Facial features were obscured to provide additional privacy protection. Written informed consent for publication of the image was obtained from the participant.

Supplementary Figure 3 Choice reaction test.

Supplementary Figure 4 Modified Agility T-test.
